# Accumulation of TDP-43 causes karyopherin-α4 pathology that characterises amyotrophic lateral sclerosis

**DOI:** 10.3389/fnins.2025.1558227

**Published:** 2025-07-23

**Authors:** Manpreet Singh Atwal, Jerneja Nimac, Urša Čerček, Sarah Ricarda Goesch, Hannah Rebecca Goesch, Paraskevi Tziortzouda, Tiziana Ercolani, Anna Zatorska, Terouz Pasha, Ivo Carre, Jacqueline Mitchell, Claire Troakes, Bart Tummers, Vera Župunski, Boris Rogelj, Tibor Hortobágyi, Frank Hirth

**Affiliations:** ^1^Department of Basic and Clinical Neuroscience, Maurice Wohl Clinical Neuroscience Institute, Institute of Psychiatry, Psychology and Neuroscience, King's College London, London, United Kingdom; ^2^Department of Biotechnology, Jozef Stefan Institute, Ljubljana, Slovenia; ^3^Graduate School of Biomedicine, Faculty of Medicine, University of Ljubljana, Ljubljana, Slovenia; ^4^London Neurodegenerative Diseases Brain Bank, Department of Basic and Clinical Neuroscience, Institute of Psychiatry, Psychology and Neuroscience, King's College London, London, United Kingdom; ^5^Centre for Inflammation Biology and Cancer Immunology, King’s College London, London, United Kingdom; ^6^Faculty of Chemistry and Chemical Technology, University of Ljubljana, Ljubljana, Slovenia; ^7^Department of Neurology, University of Debrecen, Debrecen, Hungary; ^8^Centre for Healthy Brain Ageing, Institute of Psychiatry, Psychology and Neuroscience, King's College London, London, United Kingdom

**Keywords:** amyotrophic lateral sclerosis, TDP-43, C9ORF72, karyopherin, KPNA4, nuclear import

## Abstract

Cytoplasmic mislocalisation and nuclear depletion of TDP-43 are pathological hallmarks of amyotrophic lateral sclerosis (ALS), including mutations in the *C9ORF72* gene that characterise the most common genetic form of ALS (C9ALS). Studies in human cells and animal models have associated cytoplasmic mislocalisation of TDP-43 with abnormalities in nuclear transport receptors, referred to as karyopherins, that mediate the nucleocytoplasmic shuttling of TDP-43. Yet the relationship between karyopherin abnormalities and TDP-43 pathology are unclear. Here we report karyopherin-α4 (KPNA4) pathology in the spinal cord of TDP-43-positive sporadic ALS and C9ALS patients. Structural analyses revealed the selective interaction between KPNA subtypes, especially KPNA4, with the nuclear localisation signal (NLS) of TDP-43. Targeted cytoplasmic mislocalisation and nuclear depletion of TDP-43 caused KPNA4 pathology in human cells. Similar phenotypes were observed in *Drosophila* whereby cytoplasmic accumulation of the TDP-43 homolog, TBPH, caused the nuclear decrease and cytosolic mislocalisation of the KPNA4 homolog, Importin-α3 (Impα3). In contrast, induced accumulation of Impα3 was not sufficient to cause TBPH mislocalisation. Instead, targeted gain of Impα3 in the presence of accumulating cytosolic TBPH, restored Impα3 localisation and partially rescued nuclear TBPH. These results demonstrate that cytoplasmic accumulation of TDP-43 causes karyopherin pathology that characterises ALS spinal cord. Together with earlier reports, our findings establish KPNA4 abnormalities as a molecular signature of TDP-43 proteinopathies and identify it as a potential therapeutic target to sustain nuclear TDP-43 essential for cellular homeostasis affected in ALS and frontotemporal dementia.

## Introduction

Amyotrophic lateral sclerosis (ALS) is the third most common neurodegenerative disorder characterised by the progressive degeneration of upper and lower motor neurons in the brain and spinal cord (Hardiman et al., 2017; Hortobagyi and Cairns, 2017; Renton et al., 2014). ALS shares several clinico-pathological features with frontotemporal dementia (FTD) (Lomen-Hoerth et al., 2002; Van Langenhove et al., 2012), including the accumulation and aggregation of aberrant, intracellular inclusions of TAR DNA-binding protein 43 (TDP-43) (Arai et al., 2006; Neumann et al., 2006) that are found in 97% of ALS and 45% of FTD cases (Ling et al., 2013).

TDP-43 is an evolutionarily conserved DNA–RNA binding protein, encoded by the *TARDBP* gene (Ayala et al., 2005). The ubiquitously expressed protein is composed of two RNA recognition motifs (RRM1 and RRM2), a bipartite nuclear localisation signal (NLS), and a nuclear export signal (NES) alongside a glycine-rich C-terminal low complexity domain, although the presence of the NES is disputed (Tziortzouda et al., 2021). TDP-43 typically resides in the nucleus but can shuttle between the nucleus and cytoplasm to exert its multiple functions that include splicing regulation as well as trafficking and stabilisation of RNA (Buratti and Baralle, 2008; Tziortzouda et al., 2021). Mutations in *TARDBP* have been found to characterise a small number of familial forms of ALS (Sreedharan et al., 2008; Kabashi et al., 2008; Yokoseki et al., 2008) which further emphasises a direct role of TDP-43 pathology in disease onset and progression. Pathological cytoplasmic TDP-43 inclusions concur with its nuclear depletion, suggesting a cellular state whereby both nuclear loss and cytoplasmic gain of TDP-43 function contribute to disease onset and progression (Winton et al., 2008; Igaz et al., 2011; Walker et al., 2015; Solomon et al., 2018).

TDP-43 pathology is associated with almost all cases of ALS/FTD characterised by a hexanucleotide GGGGCC (G_4_C_2_) repeat expansion in intron 1 of chromosome 9 open reading frame 72 (*C9ORF72*), the most common genetic cause of ALS and FTD (C9ALS/FTD) (DeJesus-Hernandez et al., 2011; Renton et al., 2011; Murray et al., 2011). These cases are also characterised by the accumulation of toxic dipeptide-repeat proteins (DPRs) generated through repeat-associated non-ATG translation of the sense and antisense G_4_C_2_ repeat RNA (Mori et al., 2013). Several studies have identified deficits in nucleocytoplasmic transport (NCT) and nuclear pore complex (NPC) as critical events in C9ALS/FTD (Freibaum et al., 2015; Zhang et al., 2015; Jovicic et al., 2015; Boeynaems et al., 2016a,b), including pathological abnormalities of nuclear transport receptors termed karyopherins (Freibaum et al., 2015; Jovicic et al., 2015; Lee et al., 2016; Chou et al., 2018; Solomon et al., 2018).

Karyopherins are classified into alpha (KPNA) and beta (KPNB) families (Pasha et al., 2021), with KPNAs interacting with TDP-43 (Nishimura et al., 2010; Freibaum et al., 2015; Chou et al., 2018). KPNA proteins comprise three functional domains: the N-terminal importin-B-binding domain, a C-terminal CAS domain and ten helical armadillo (ARM) repeat motifs which facilitate NLS binding of nuclear cargo (Pasha et al., 2021). In addition to their role in nucleocytoplasmic transport, karyopherins have been recognised for their functions as molecular chaperones and disaggregases (Guo et al., 2018; Gasset-Rosa et al., 2019; Hutten et al., 2020; Khalil et al., 2022). Moreover, karyopherin abnormalities have been implicated in TDP-43 pathology with disrupted karyopherin-binding been shown to disturb TDP-43 nucleocytoplasmic transport (Tziortzouda et al., 2021). Studies using *Drosophila* models of C9ALS/FTD identified a vicious feedback cycle for DPR-mediated TDP-43 proteinopathy characterised by cytoplasmic TDP-43 and KPNA4 mislocalisation (Solomon et al., 2018), a phenotype that was also observed in post-mortem frontal cortex tissue of C9FTD and sporadic FTD-TDP cases (Solomon et al., 2018; Chou et al., 2018). While these studies established a direct link between TDP-43 and KPNA4 pathology in FTD, it has remained elusive whether these karyopherin abnormalities also characterise ALS and whether they are the cause or consequence of TDP-43 pathology.

## Materials and methods

### Human post-mortem tissue

Human post-mortem spinal cord samples were provided by the London Neurodegenerative Diseases Brain Bank (King’s College London, UK). Consent for autopsy, neuropathological assessment and research was obtained for all donors and all studies were carried out under the ethical approval of the tissue bank (08/MRE09/38 + 5 and 18/WA/0206). Block taking for histological and immunohistochemical analyses and neuropathological assessment was performed in accordance with published guidelines (Cairns et al., 2007). Control cases were defined as age matched subjects with no clinical history or neuropathological evidence of a neurodegenerative condition (showing age-related pathology only) (see Table 1). All sporadic ALS cases showed typical TDP-43 pathology in the form of neuronal cytoplasmic inclusions and additional glial inclusions in some cases. The C9ALS cases had all previously undergone repeat primed PCR to identify the repeat expansion, and all showed the characteristic TDP-43 and p62 pathology associated with C9ORF72 expansion (Al-Sarraj et al., 2011).

**Table 1:** Analysis of KPNA4 pathology in post-mortem human spinal cord.

Case number	Sex	Age	PMD	Neuro-pathological diagnosis	Overall motor neuron staining intensity	Neuronal nuclear staining	Neuronal cytoplasmic staining	Reduced nuclear staining
Control								
BBN_22594	F	77	21	Control	2	1	2	Moderate
BBN_10209	M	63	23	Control	1	1	0	No
BBN_16277	M	54	30	Control	2	3	1	No
BBN_22991	F	73	27	PART tau stage 1	1	1	0	No
BBN_10992	M	65	26	PART tau stage 1	2	2	1	No
sALS								
BBN002.26993	M	69	52.5	ALS with extensive extramotor TDP-43 pathology	2	1	2	Moderate
BBN_22631	F	73	70	ALS with limbic TDP-43 pathology	1	0	2	Severe
BBN_15192	M	55	4.5	ALS with amygdala TDP-43 pathology	1	1	2	Moderate
BBN_24296	M	68	73	FTLD-ALS	1	1	2	Moderate
BBN_19699	F	50	56	ALS	1	1	2	Moderate
BBN002.28672	F	54	67.5	ALS with extensive extramotor TDP-43 pathology	1	1	2	Moderate
BBN_10210	M	54	69	ALS with limbic TDP-43 pathology	1	1	2	Moderate
BBN002.26162	F	68	51.5	ALS	1	1	3	Moderate
C9ALS								
BBN_6254	M	53	82	C9FTLD-ALS	1	0	2	Severe
BBN_16380	F	59	34.5	C9ALS	1	1	3	Severe
BBN_21794	F	59	74	C9ALS	2	1	3	Severe
BBN_10306	M	64	68	C9ALS	2	0	3	Severe
BBN_6227	M	55	76	C9ALS	1	0	3	Severe
BBN_16304	M	59	46	C9FTLD-ALS	1	0	2	Severe
BBN_6252	F	43	69	C9ALS	2	0	3	Severe
BBN_6242	F	39	69.5	C9ALS	1	1	2	Moderate
BBN_6251	M	62	73.5	C9ALS	1	1	2	Moderate

Study number, sex, age (years), post-mortem delay (PMD, hours), neuropathological diagnosis. KPNA4-stained spinal cord samples were assessed and scored based on staining intensity: 0 - none, 1 - mild, 2 - moderate, 3 - intense; reduced nuclear staining defined as the difference between neuronal nuclear and cytoplasmic staining. PART, primary age-related tauopathy; ALS, amyotrophic lateral sclerosis; FTLD, frontotemporal lobar degeneration.

### Human post-mortem tissue immunohistochemistry

For immunohistochemistry of post-mortem human spinal cord samples 7 μm thick sections were prepared from formalin-fixed, paraffin–embedded tissue blocks. To perform immunostaining, paraffin was removed with xylene and sections were rehydrated in an ethanol series (99 and 95%) for 3 min each. Slides were then microwaved in sodium citrate buffer to enhance antigen retrieval. Non-specific binding was blocked for 20 min with Normal Swine Serum (NSS) (Agilent) at 1:10 in TBS. For immunohistochemistry, primary rabbit anti-KPNA4 (1:500, Novus) was added together with NSS at 1:100 in TBS and incubated overnight at 4°C. After washing with TBS, sections were incubated with biotinylated secondary antibody (Agilent) diluted 1:100 in TBS, followed by incubation with ABC HRP (Thermo Fisher Scientific) solution. Finally, sections were incubated for up to 10 min with 3,3′-diaminobenzidine (DAB) chromogen (Sigma-Aldrich, Dorset UK) in TBS containing 0.04% hydrogen peroxide. Sections were counterstained with Harris’ haematoxylin and dehydrated in IMS and xylene. Finally, sections were coverslipped using Ralmount glue and allowed to set.

### Semi-quantitative evaluation of KPNA4 pathology in human post-mortem spinal cord

For each human post-mortem case (Control: *n* = 5, sALS n = 8, C9ALS: *n* = 9) a minimum of 25 anterior horn motor neurons were assessed on a minimum of 3 consecutive sections with anti-KPNA4 immunohistochemistry. Immunostaining was examined and assessed by a consultant neuropathologist (TH) blind to diagnosis. A variety of indicators reflecting KPNA4 morphology, location and intensity were quantified and semi-quantitatively scaled using an Olympus Viewer microscope. The overall motor neuronal, cytoplasmic and nuclear staining intensity, respectively, was scored (0 = none, 1 = mild, 2 = moderate, 3 = intense); reduced nuclear staining defined as the severity of reduced neuronal nuclear staining as compared to cytoplasmic staining (no difference, mild, moderate, severe reduction).

### Structural and computational analysis of KPNA and TDP-43 protein interaction

For computational interaction analyses, structural data available at the Protein Data bank^1^ were used for KPNA1 (PDB: 6WX9); KPNA2 (7N9H); KPNA4 (PDB: 5XZX); KPNA6 (PDB: 4UAD); together with the TDP-43 N-terminal domain (PDB: 5MRG) and its Alphafold-predicted full-length structure (AF-Q13148-F1, available at https://alphafold.ebi.ac.uk/entry/Q13148). Subsequent docking computations between TDP-43 and KPNA proteins were performed with all ligands, water molecules and homo-oligomers stripped using the PyMOL software^2^ and compared against the established KPNA2-NLS-TDP-43 complex (Doll et al., 2022) as quality control.

To accommodate highly dynamic interactions between TDP-43 NLS and KPNA proteins, the CABS-DOCK server^3^ was used which utilizes a coarse-grained peptide protein docking approach to allow for free conformational sampling (Kurcinski et al., 2015; Blaszczyk et al., 2019). For each KPNA complex, the peptide interaction with the highest conformational and spatial agreement to the experimentally determined structure of KPNA2-NLS-TDP-43 (Doll et al., 2022) was chosen. These complexes were analysed for accurate anti-parallel bipartite binding of the NLS of TDP-43 (K^82^RKMDETDASSAVKVKR^98^) to the major and minor groove of KPNA via its bipartite motive, in addition to engagement of highly conserved KPNA Asparagine and Tryptophan residues.

To confirm the NLS conformational and spatial affinity towards the investigated KPNA proteins, CABS-DOCK binding predictions were validated using the HPEP-DOCK webserver,^4^ a global hierarchical docking algorithm for blind protein-peptide docking, (Zhou et al., 2018). From the resulting conformations, the best model was selected based on binding accuracy between CABS-DOCK and HPEP-DOCK and visualized using PyMOL. Docking models were energy minimized using the YASARA program (http://www.yasara.org) utilizing physical parameters set out by Krieger et al. (2009).

To determine the binding affinity of the different complexes, their energy minimized PDB structures were uploaded to the PROtein binDIng enerGY prediction (PRODIGY) protein–protein algorithm, accessible via the PRODIGY webserver,^5^ using the default settings (Xue et al., 2016). This yielded the calculated estimated values for the total free energy of binding [DG (kcal/mol)] and the dissociation constant [Kd (M)]. In addition, the energetical contribution from the different binding interactions was calculated.

To calculate the molecular contact surface area between the predicted, energy minimized complexes and the experimental KPNA-NLS complex (PDB: 7N9H), all PDB structures were uploaded into the YASARA program^6^ using the “Surface area between molecules” application and selected to probe for all atoms on the molecular surface area of the KPNA proteins, whose Van-der-Waals radius subtracted from the distance to any atom of the NLS is smaller than 2.0 Å (Xue et al., 2016). Differences in binding interactions between the investigated KPNA proteins and the TDP-43 NLS were analyzed using *BIOVIA Discovery Studio* Visualizer^7^ and the generated interaction maps visualized with Biorender.^8^

### *In vivo* interaction between karyopherin-*α* and TDP-43

cDNA of TDP-43-wt and ∆NLS-TDP-43 were inserted into vector pcDNA3.1-myc-BioID2-MCS (Addgene, # 74223), containing coding sequence for biotin ligase (BioID2) and the whole constructs were then cloned into pcDNA5-FRT vector to establish Flp-In HEK293 cell lines. Primer sequences used for molecular cloning of constructs with BioID2 ligase were:

**Table:** 

Oligonucleotide sequences and template DNA	Construct
5’-ACTAGTGGATCCGATGTCTGAA-3′ 5’-CTTGGTACCGAGCTCTACATT-3’ DNA: pEGFP-C1-TDP-43wt	TDP-43wt amplification for insertion into the pcDNA3.1-myc-BioID2-MCS vector
5’-CGTGGATCCGATGTCTGAATATATTCGGG-3′ 5’-AGAGGTACCCTAAGCGTAATCTGGA-3’ DNA: pEGFP-C1-TDP-43∆NLS	∆NLS-TDP-43 amplification for insertion into pcDNA3.1-myc-BioID2-MCS
5’-CAAGAAGCTTGGTACCGAG-3′ 5’-GTGGCTAGCCAGCTTAAGTTTAAACGCTAGAGTCC-3’ DNA: pcDNA5-FRT	Inverse amplification of the pcDNA5-FRT vector and the addition of *NheI* restriction site, which allows the insertion of transcripts for fusion proteins with BioID2 ligase.
5’-TCCGGTACCATGGAACAAAAACTC-3′ 5’-CCGAAGCTTCTAGCTTCTTCTCAGGCTGAA-3’ DNA: pcDNA3.1-myc-BioID2-MCS	Amplification of the BioID2 adding *KpnI* and *HindIII* restriction sites that allow cloning of biotin ligase fusion proteins at its C-terminal.

Flp-In HEK293 cells containing FRT sites for preparation of stable cell lines were maintained in DMEM culture medium supplemented with 10% tetracycline-free FBS, 1% mixture of penicillin/streptomycin and 100 μg/mL Zeocine (InvivoGen). The cells were grown at 37°C in a humidified atmosphere with 5% CO_2_. Stable cell lines were prepared according to previously established protocol from ThermoFisher Scientific. Cells were co-transfected with pcDNA5-FRT plasmids (pcDNA5-FRT-myc-BioID2 or pcDNA5-FRT-myc-BioID-TDP-43wt or pcDNA5-FRT-myc-BioID-∆NLS-TDP-43) and pOG44 plasmids with Lipofectamine 2000 reagent (Invitrogen). Transfected cells were cultured for 48 h before adding a selection medium containing hygromycin B Gold (175 μg/mL, InvivoGen). Selection medium was changed twice per week until a resistant population was obtained.

BioID2 experiments were performed based on a modified version of a previously established protocol (Božič et al., 2022). Primary antibodies used were: KPNA1 (mouse, Santa Cruz Biotechnology, sc-101292, 1:750), KPNA3 (mouse, Santa Cruz Biotechnology, sc-514101, 1:750), KPNA4 (rabbit, Proteintech, 12,463-AP, 1:750), KPNA6 (mouse, Santa Cruz Biotechnology, sc-390055, 1:750), KPNA7 (rabbit, GeneTex, GTX31991, 1:1,000), TDP-43 (goat, Abcam, ab80608, 1:100) KPNA4 (mouse, SCBT, sc-390535, 1:50), Myc (rabbit, Proteintech, 60,003-2-Ig, 1:250) and Streptavidine-Atto488 (Rockland laboratories, S000-52). Secondary antibodies used were anti-rabbit-HRP (Jackson ImmunoResearch, 111–035-045, 1:5,000 in blocking solution) or anti-mouse-HRP (Jackson Immunoresearch, 115–035-068, 1:5,000 in blocking solution), anti-mouse Alexa488 (1:1,000), anti-rabbit Alexa 647 (1:1,000), anti-goat Alexa 555 (1:1,000) all from Cell Signaling.

#### Immunocytochemical analysis of KPNA4 and TDP-43 protein interaction

cDNA of mScarletI was inserted into pcDNA5 FRT TO with TDP-43-wt or ∆NLS-TDP-43 to establish Flp-In HEK293 cell lines. Primer sequences used for molecular cloning of constructs with mScarletI were:

**Table:** 

Oligonucleotide sequences and template DNA	Construct
5′- AGCTCCGGAGAACAGAAGCT −3′ 5’-CGAGCTCGGTACCAAGCT-3’ DNA: pcDNA5-APEX-myc-TDP-43wt	Amplification of the pcDNA5-FRT vector with myc-TDP-43-wt.
5′- AGCTCCGGAGAACAGAAGCT −3′ 5’-CGAGCTCGGTACCAAGCT-3’ DNA: pcDNA5-APEX-myc-TDP-43∆NLS	Amplification of the pcDNA5-FRT vector with myc-∆NLS-TDP-43.
5’-AAACTTAAGCTTGGTACCGAGCTCGATGGTGAGC AAGGGCGAGG-3′ 5’-TGATCAGCTTCTGTTCTCCGGAGCTCTTGTACAG CTCGTCCATGC-3’ DNA: pcDNA5-mScarletI-myc	mScarletI amplification with complementary sequence for annealing with amplified pcDNA5-FRT myc-TDP-43-wt or pcDNA5-FRT myc-∆NLS-TDP-43

Flp-In HEK293 cells containing mScarletI-myc-TDP-43-wt or mScarletI-myc-∆NLS-TDP-43 or mScarletI-myc were established as described in *In vivo interaction between Karyopherin-α and TDP-43*. Cells were co-transfected with pcDNA5-FRT plasmids (pcDNA5-FRT-mScarletI-myc or pcDNA5-FRT-mScarletI-myc-TDP-43wt or pcDNA5-FRT-mScarletI-myc-∆NLS-TDP-43) and pOG44 plasmids with Lipofectamine 2,000 reagent (Invitrogen).

Cells were grown on poly-L-lysine-coated coverslips. Twenty-four hours after induction of gene expression with doxycycline, the cells were fixed with 4% paraformaldehyde in PBS. After three washes, blocking and permeabilisation was performed in a blocking solution (5% goat serum (Euroclone) in PBS, with 0.1% Tween-20). The coverslips were then incubated with the primary antibodies in the blocking solution for 1 h at room temperature. After washing three times in PBS, the coverslips were incubated with the secondary antibodies in blocking solution for 1 h at room temperature. After further washing steps, the nuclei were stained with DAPI and mounted on glass slides using ProLong Gold (Thermo Fisher Scientific). Primary antibodies used were: TDP-43 (rabbit, Proteintech, 12,892-1-AP, 1:1,000) KPNA4 (mouse, SCBT, sc-390535, 1:50). Secondary antibodies used were anti-mouse Alexa488 (1:1,000), anti-rabbit Alexa 647 (1:1,000) all from Cell Signaling.

#### *Drosophila* stocks and genetics

All fly stocks were maintained at 25°C in a 12-h light–dark cycle on standard cornmeal food. The *UAS-Kap-α3* (Importin-*α3, Impα3*) stock was generously provided by Herve Tricoire (Paris Diderot University) (Sowa et al., 2018). The *FKH-Gal4* stock targeting Gal4 to salivary gland cells was generously provided by Eric Baehrecke (University of Massachusetts Medical School, Worcester) (Velentzas et al., 2018). The *w^1118^* line was obtained from the Bloomington *Drosophila* Stock Center. The *ΔNLS-TBPH* line expresses the *Drosophila* TDP-43 homolog TAR DNA-Binding Protein-43 Homologue, TBPH with a mutated nuclear localisation signal (ΔNLS) under the control of the endogenous *TBPH* promoter and has been described previously (Solomon et al., 2018).

#### *Drosophila* immunohistochemistry

Salivary gland preparations were dissected and stained according to protocols previously described (Solomon et al., 2018). Larval salivary glands were immunolabelled using rabbit anti-TBPH (1:2,000) (Diaper and Hirth, 2013), rabbit anti-Importin-α3 (1:300, kind gift from S. Cotterill) and mouse monoclonal anti-MAB414 (1:500, Abcam). Secondary antibodies conjugated to Alexa Fluor^®^ 488 and 568 (Life Technologies) were used at a final concentration of 1:150.

#### Confocal microscopy, image acquisition, and analysis

All images were obtained using a Nikon A1R confocal microscope driven by NIS-Elements AR software and equipped with a 60×, NA 1.4 objective or Zeiss LSM 710 inverted confocal laser scanning microscope with a Plan-Apochromat 63 × and 1.4 NA M27 oil immersion objective using immersion oil (Carl Zeiss). Identical confocal microscope setting was used when imaging both control and experimental genotypes. For quantification of the nuclear/cytoplasmic ratios, full Z-stacks were taken at 0.5 μm intervals across the plane of salivary gland cells. The nuclear region was defined by immunolabelling with the nuclei marker DAPI, and MAB414, which aided in determining TBPH/Importin-α3 localisation. Images were processed using ImageJ, with nuclear-cytoplasmic ratios established from pixel intensities that were used as a quantitative readout for protein expression either side of the nuclear membrane.

#### Statistical analysis

Statistical analysis was performed using GraphPad Prism 8.0. Variability of values is given as the standard error of the mean (SEM). Data distributions were tested for normality using the Shapiro–Wilk normality test. In case of non-parametric distribution Mann–Whitney test was applied to compare two groups. A one-way ANOVA was used for a comparison of means with multiple experimental conditions. Tukey’s *post-hoc* test was used to assess statistical significance of KPNA4 gain-of-function experiments. Nuclear-cytoplasmic ratios between *w^1118^* and ΔNLS-TBPH larvae were assessed by unpaired *t*-tests. *p* < 0.05 was considered significant.

## Results

### KPNA4 pathology in ALS patient spinal cord

To determine whether KPNA4 pathology characterises ALS, human post-mortem samples of TDP-43-positive sporadic ALS and C9ALS patient cases together with healthy, age-matched controls were probed for KPNA4. We determined the cellular localisation of KPNA4 using DAB conjugated immunohistochemistry of KPNA4 performed on 22 post-mortem spinal cord tissue sections obtained from 5 control cases (2 females and 3 males; mean ± SD age 66.4 ± 9 years), 8 sporadic ALS cases (4 females and 4 males; mean ± SD age 61.4 ± 9 years) and 9 C9ALS cases (4 females and 5 males; mean ± SD age 73.7 ± 10.8 years). Samples were scored based on a semi-quantitative scale for anti-KPNA4 immunoreactivity to assess overall motor neuron staining, neuronal nuclear staining, the severity of reduced nuclear staining and neuronal cytoplasmic staining, the findings of which are summarised in Table 1.

**Figure 1:**
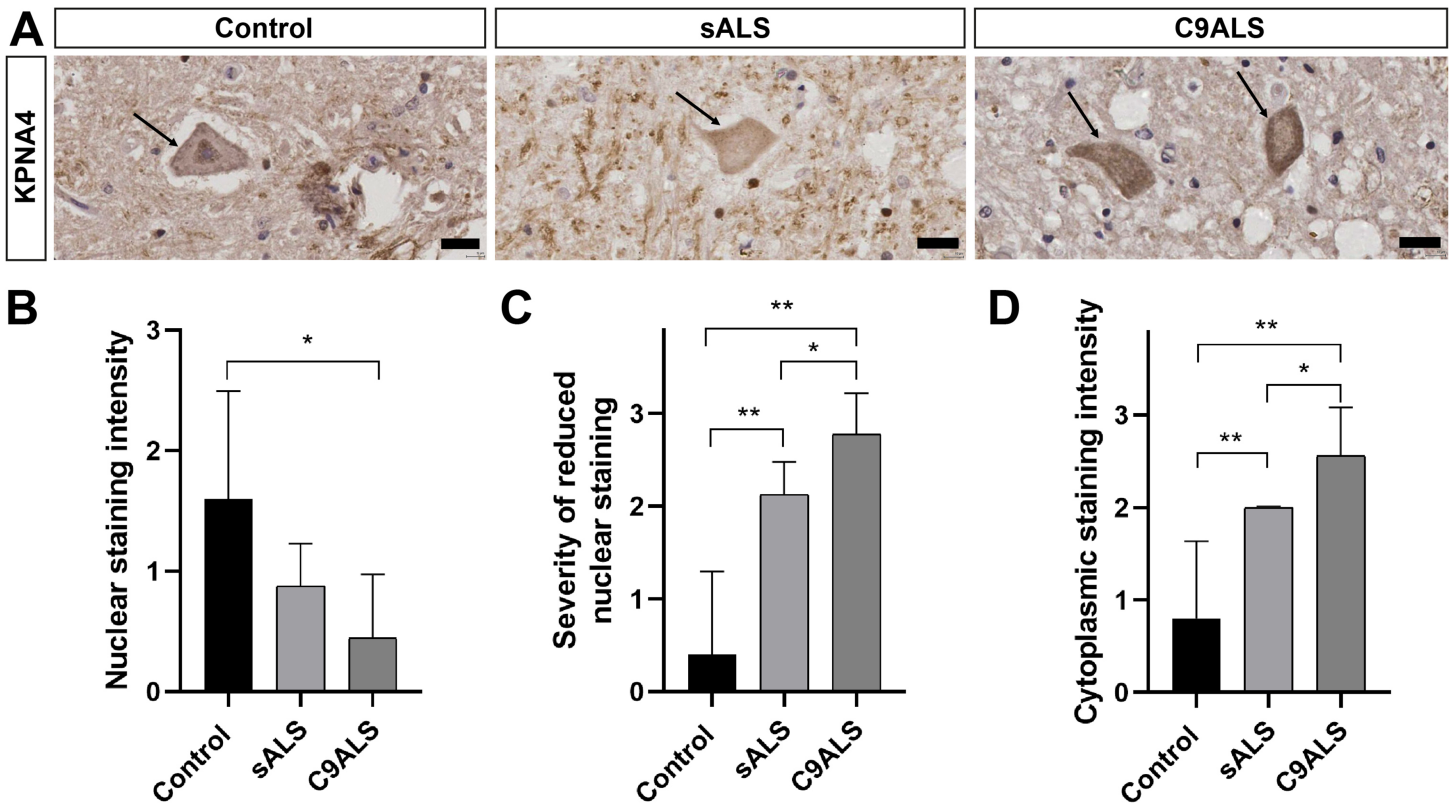
KPNA4 pathology in ALS patient spinal cord. **(A)** DAB conjugated immunohistochemistry of KPNA4 in spinal motor neuron (arrow) of control, sporadic ALS (sALS) and C9ALS cases. KPNA4 immunolabelling reveals nuclear staining with minimal cytoplasmic labelling in control, however nuclear decrease and cytoplasmic accumulation of KPNA4 can be detected in spinal motor neurons (arrows) of sporadic ALS and C9ALS cases. Note, dystrophic neurites or inclusions were not detected by KPNA4 immunohistochemistry in all spinal cord samples; scale bars: 20 μm. **(B)** Decrease in anti-KPNA4 nuclear staining intensity in sporadic ALS and C9ALS as compared to control cases (*p < 0.05). **(C)** Severity of reduced nuclear KPNA4 immunostaining is significantly increased in sALS and C9ALS (*p < 0.05; **p < 0.01). **(D)** Significantly increased cytoplasmic localisation of KPNA4 in sALS and C9ALS cases, compared to control. For each human post-mortem case (control, *n* = 5; sALS, *n* = 8; C9ALS, *n* = 8) a minimum of 25 anterior horn motor neurons were assessed and scored (see Table 1) on a minimum of 3 consecutive sections with anti-KPNA4 immunolabelling. Statistical analyses were performed using one-way ANOVA with Tukey’s multiple comparison post-hoc test; *p < 0.05; **p < 0.01; mean ± SEM shown. Scale bars, 20 μm.

KPNA4 immunoreactivity in control cases predominantly displayed nuclear distribution with minimal cytoplasmic immunostaining (Figure 1A). In comparison, immunohistochemical analysis revealed a decrease in nuclear KPNA4 expression in both sporadic ALS and C9ALS (Figure 1B), which was markedly pronounced in cases of C9ALS (Figure 1C). In addition to its nuclear decrease, increased cytoplasmic expression of KPNA4 was detectable in C9ALS cases as recorded in the semi-quantitative assessment of neuronal cytoplasmic staining (Figure 1D). No dystrophic neurites or inclusions were observed in any post-mortem spinal cord samples. Together, these data identify KPNA4 pathology in TDP-43-positive ALS patient post-mortem spinal cord. The observed KPNA4 pathology is characterised by reduced nuclear abundance and increased cytoplasmic localisation in sporadic ALS cases, a phenotype that is further pronounced in C9ALS.

### Interaction of TDP-43 and KPNA proteins

A pathological hallmark of ALS is the cytoplasmic accumulation and nuclear depletion of TDP-43 (Tziortzouda et al., 2021). The observed KPNA4 phenotype may suggest that KPNA4 and TDP-43 pathologies could be interlinked. To investigate their possible interrelation, we first examined TDP-43 and KPNA interaction under physiological conditions. Previous studies have shown that TDP-43 interacts with KPNA via its NLS essential for nuclear transport (Nishimura et al., 2010; Winton et al., 2008; Igaz et al., 2011; Walker et al., 2015; Solomon et al., 2018). We confirmed this interaction using a proximity biotinylation assay utilizing biotin ligase 2 (BioID2) in human Flp-In HEK293 cell lines expressing control BioID2, or human TDP-43 in either its wildtype form (BioID2-Myc-TDP-43) or with its NLS deleted (BioID-Myc-TDP-43-ΔNLS) (Supplementary Figures S1A,B). Subsequent pulldown using BioID2 as bait followed by immunostaining revealed interaction between KPNA4 and TDP-43 that was dependent on its nuclear localisation signal (Supplementary Figures S1C,D). We performed similar pulldown experiments with a selection of other KPNAs and detected a TDP-43-NLS dependent interaction with KPNA1 and KPNA3, but not with KPNA6 (Supplementary Figures 1C,D), despite a robust signal detection in whole cell lysate for all KPNAs tested.

To gain mechanistic insight into these interactions, a CABS- and HPEP-DOCK computational binding analysis was carried out. CABS-DOCK utilizes a coarse-grained peptide protein docking approach to allow for free conformational sampling (Kurcinski et al., 2015; Blaszczyk et al., 2019), while the global hierarchical docking algorithm HPEP-DOCK allows for blind protein-peptide docking (Zhou et al., 2018). We first performed a structural and kinetic computational analysis of KPNA2 with the NLS of TDP-43 and validated this against the only experimentally determined KPNA-TDP-43 structure of KPNA2 (Doll et al., 2022) (Supplementary Figures S2A–C; Supplementary Table S1). We applied a dynamic docking approach by excluding the rigidly structured, NLS-flanking NTD and RRM1 domains, which minimised possible interference during the modelling process. Both CABS- and HPEP-DOCK computational models demonstrated comparable biophysical interactions that closely aligned with the experimental data established for KPNA2 (Supplementary Figure S2).

**Figure 2:**
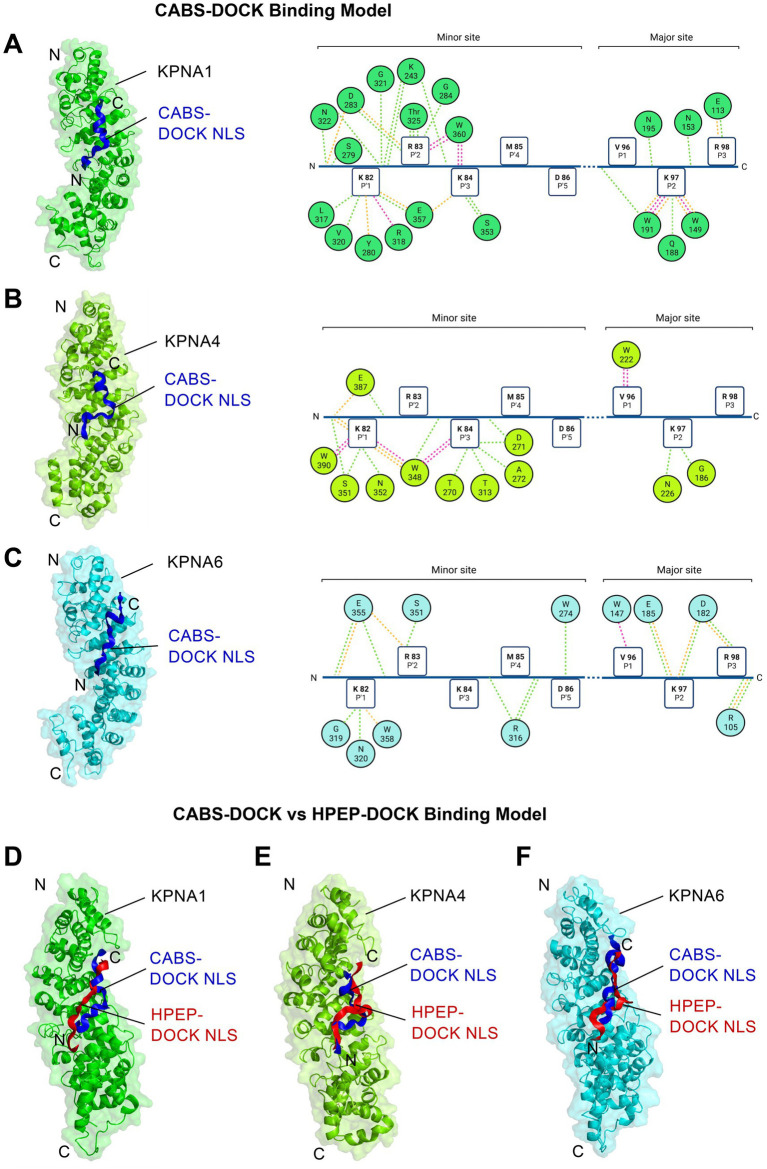
Structural analysis of KPNA binding to TDP-43-NLS. Computational CABS-DOCK analysis reveals TDP-43 nuclear localisation signal (NLS, blue) and KPNA binding (left) together with corresponding molecular contact maps (right) for **(A)** KPNA1 (PDB: 6WX9, green), **(B)** KPNA4 (PDB: 5XZX, chartreuse) and **(C)** KPNA6 (PDB: 4UAD, cyan). The corresponding 2D contact map shows the intermolecular interaction between the minor and major binding site of the NLS (squares) and the active residues of the KPNA proteins (coloured spheres) which is mediated via conserved Tryptophan (W) residues. Interaction types are, electrostatic and salt-bridge (orange), hydrogen bonds (green), hydrophobic contact (pink). Note, all models generated reveal the bipartite binding pattern, with the N-terminal P1´- P5´ residues (KRKMD) interfacing with the minor groove residues (ARM6-8) of the KPNA protein, and the C-terminal P1’ – P5’ residues (VKRAV) dovetailing with the major groove (ARM2-4). **(D–F)** Comparison between CABS-DOCK (blue peptide) and HPEP-DOCK (red peptide) computational models reveals correspondences for binding prediction between TDP-43-NLS and **(D)** KPNA1, **(E)** KPNA4 or **(F)** KPNA6.

We then performed conformational analyses of KPNA1 and KPNA4 protein structures, which bound TDP-43 in pull-down experiments, together with KPNA6 for which we did not observe BioID-related binding (Supplementary Figure S1C). This revealed antiparallel interaction of the NLS with conserved tryptophan residues on the third alpha-helical (H3) structural repeat of each KPNA ARM domain (Figures 2A–C). For the KPNA1-NLS and KPNA4-NLS models, we found stronger binding to the KPNA minor site (Figures 2A,B). An exception was the KPNA6-NLS interaction, which showed slightly stronger binding at the major site (Figure 2C). The molecular contact area of each model was calculated with the KPNA1-NLS complex providing the most extensive contact (1226.6 Å^2^), followed by KPNA4-NLS (994.4 Å^2^) and KPNA6-NLS (916.5 Å^2^) (Supplementary Table 2). Analysis of the various molecular interactions identified the KPNA1-NLS complex with the most comprehensive intermolecular interaction profile, with a total number of 61 interactions. This is followed by the KPNA4-NLS complex with 39, and the KPNA6-NLS complex with the fewest intermolecular interactions of 37 (Supplementary Table S2). Using the PROtein binDIng enerGY prediction (PRODIGY) protein–protein algorithm (Xue et al., 2016), we calculated estimated values for the total binding energy ΔG (kcal/mol) of each docked KPNA-TDP-43 NLS interaction. Our data indicate KPNA1 and KPNA4 have the strongest affinity for NLS-TDP-43 with the KPNA1 and KPNA4-NLS complex possessing a binding energy of −11.5 and −11.4 kcal/mol, respectively. In comparison, KPNA6-NLS interaction had a considerably lower binding energy of −9.6 kcal/mol (Supplementary Table 2). Notably, both CABS- and HPEP-DOCK computational models demonstrated comparable biophysical interactions (Figures 2D–F). Both models thus reveal a molecular framework of NLS-dependent, transient binding between TDP-43 and KPNA.

### Cytoplasmic accumulation of TDP-43 causes KPNA4 pathology

**Figure 3:**
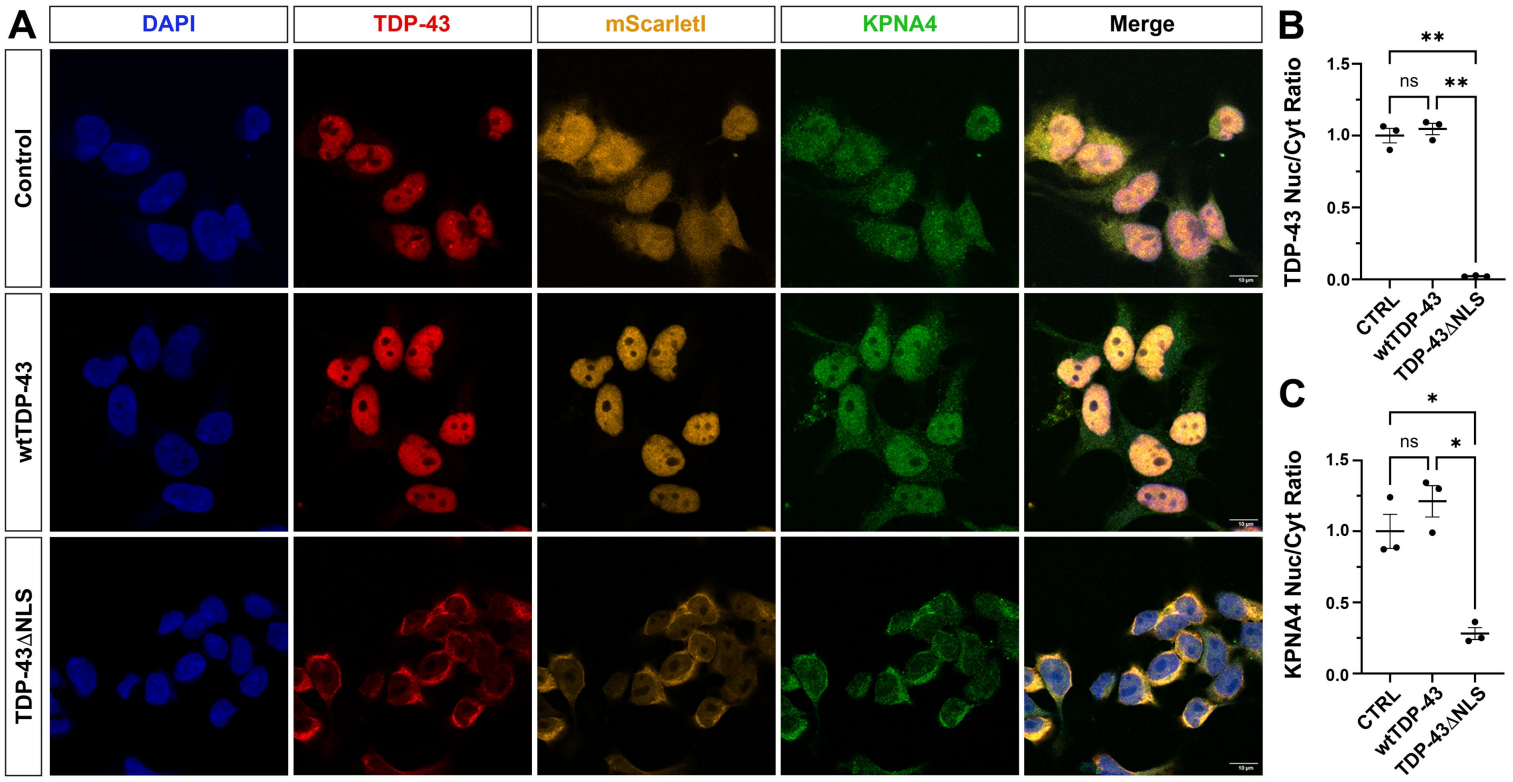
Cytoplasmic accumulation of TDP-43 causes KPNA4 pathology in human cells. **(A)** HEK293 cells expressing the reporter construct mScarletI-myc either on its own or fused to human wildtype TDP-43 (mScarletI-myc-wtTDP-43) or fused to the nuclear localisation signal depleted form (mScarletI-myc-TDP-43ΔNLS). Imaging of mScarletI and immunolabeling with anti-TDP-43 and anti-KPNA4 reveals nuclear depletion of TDP-43 and cytoplasmic accumulation of KPNA4 and TDP-43 in TDP-43-ΔNLS, but not in wildtype TDP-43 expressing cells. **(B)** Quantification of the nuclear-cytoplasmic ratio of TDP-43 in mScarletI-myc expressing control cells (CTRL); in mScarletI-myc-wt-TDP-43 expressing cells (wtTDP-43) and in mScarletI-myc-∆NLS-TDP-43 expressing cells TDP-43ΔNLS. **(C)** Quantification of the nuclear-cytoplasmic ratio of KPNA4 in CTRL, wtTDP-43 and TDP-43ΔNLS expressing cells. Examined samples sizes, mScarletI-myc cells (*n* = 831), mScarletI-myc-wt-TDP-43 cells (*n* = 826) and mScarletI-myc-TDP-43ΔNLS cells (*n* = 1,081). Statistical analyses were performed using one-way ANOVA with Tukey’s multiple comparison post-hoc test; ns, not significant; *p < 0.05; **p < 0.01; mean ± SEM shown. Scale bars, 10 μm.

To further investigate the interrelation between TDP-43 and KPNA pathologies, we made use of our BioID cell model and immunolabelled for TDP-43 and Myc (Supplementary Figure S3). We also generated a second, independent cell model expressing the reporter construct mScarletI-myc as control (CTRL), or full-length human wildtype TDP-43 fused to mScarletI-myc (mScarletI-myc-wtTDP-43), or the NLS-deleted from of human TDP-43 fused to mScarletI-myc (mScarletI-myc-TDP-43ΔNLS). Immunocytochemical analysis revealed in both cell models the cytoplasmic accumulation and near-complete nuclear depletion of TDP-43 when compared to expression of its wildtype form (Figure 3; Supplementary Figure S3). Determination of the nuclear-cytoplasmic ratio of TDP-43 revealed a statistically significant decrease in nuclear TDP-43 when depleted of its NLS when compared to wt-TDP-43 and the mScarletI-myc control (Figure 3B; Tukey *t*-test: *p* = 0.001). Of note, fusion with mScarletI had no effect on the localisation of the TDP-43 variant. mScarletI-myc was equally distributed in the nucleus and cytoplasm (Figure 3A); however, its subcellular distribution was significantly increased in the nuclei when fused to wt-TDP-43 and significantly decreased when fused to TDP-43ΔNLS, consistent with the localisation of the TDP-43 variant (Supplementary Figure S4). Immunolabelling for KPNA4 showed cytoplasmic accumulation and reduction in the nuclei in mScarletI-myc-TDP-43-ΔNLS expressing cells compared to mScarletI-myc-TDP-43 in which TDP-43 was predominantly nuclear. Quantitative evaluation of the nuclear-cytoplasmic ratio of KPNA4 in the presence of ∆NLS-TDP-43 showed a statistically significant decrease in nuclear KPNA4 compared to wt-TDP-43 and the mScarletI-myc control (Figure 3D; Tukey *t*-test: *p* = 0.02). Together these findings establish that cytoplasmic mislocalisation of TDP-43 causes KPNA4 pathology *in vitro*.

**Figure 4:**
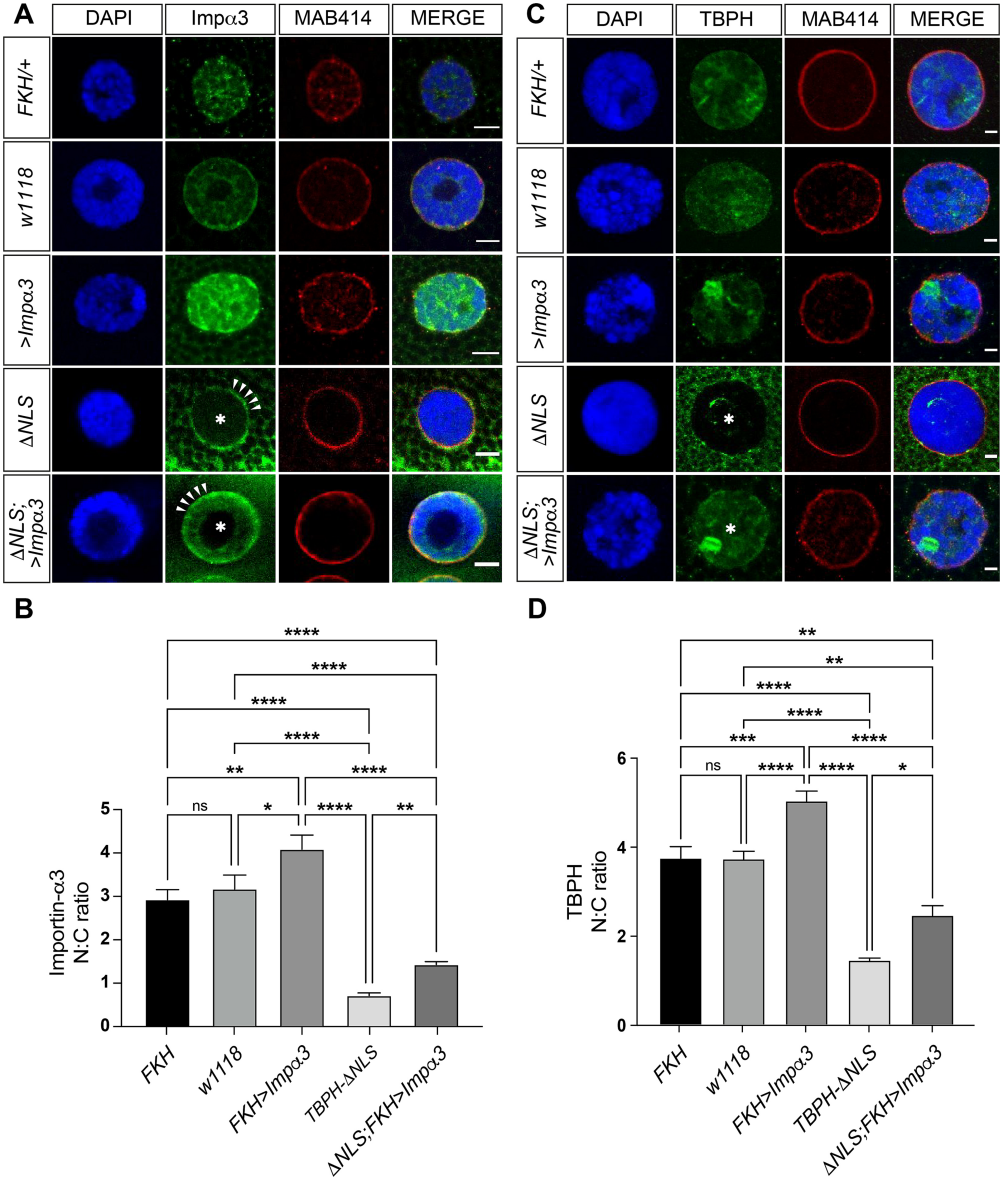
Accumulating *Impα3* does not mislocalise but partially restores nuclear TBPH. **(A)** Confocal images of L3 salivary gland cells immunolabelled with nuclear marker DAPI (blue), anti-Importin-α3 (green) and anti-MAB414 (red) detecting nuclear pore complex proteins to mark the nuclear rim. In *FKH-Gal4/+* and *w^1118^* control flies, anti-Impα3 immunostaining reveals predominantly nuclear localisation of Importin-α3. In *FKH>Impα3*, Importin-α3 labelling appears amplified in the nucleus whereas cytoplasmic accumulation of TBPH in TBPH-ΔNLS reveals nuclear depletion and cytoplasmic accumulation of Importin-α3, which is partially restored when overexpressing it in *TBPH*-*ΔNLS; FKH>Impα3,* flies. **(B)** Quantification of the nuclear-cytoplasmic ratio of Impα3 in *FKH-Gal4/+* (*n* = 4); *w^1118^* (*n* = 8); *FKH>Impα3* (*n* = 6); *TBPH-ΔNLS* (*n* = 20); *TBPH*-*ΔNLS; FKH>Impα3* (*n* = 15). **(C)** Confocal images of salivary gland cells immunolabelled with nuclear marker DAPI (blue), anti-TBPH (green) and anti-MAB414 (red). In *FKH-Gal4/+* and *w^1118^* controls, immunostaining reveals predominant nuclear localisation of TBPH. In *FKH>Impα3* flies overexpressing Importin-α3 nuclear labelling of anti-TBPH appears slightly amplified. However, in flies expressing TBPH-ΔNLS, immunostaining reveals nuclear depletion (asterisk) and cytoplasmic accumulation of TBPH. Note, overexpressing Importin-α3 in the presence of accumulating cytoplasmic TBPH (*ΔNLS; FKH>Impα3*) results in partial restoration of nuclear TBPH (asterisk). **(D)** Quantification of the nuclear-cytoplasmic ratio of anti-TBPH immunolabelling in *FKH-Gal4/+* (*n* = 27); *w^1118^* (*n* = 31); *FKH>Impα3* (*n* = 20); *TBPH-ΔNLS* (*n* = 36); *ΔNLS; FKH>Impα3* (*n* = 9). Statistical analyses were performed using one-way ANOVA with Tukey’s multiple comparison post-hoc test; ns, not significant; *p < 0.05; **p < 0.01; ***. Scale bars: A, 10 μm; C, 5 μm.

To further investigate the pathological interaction between TDP-43 and KPNA4, we used *Drosophila melanogaster* which has previously been used to investigate cellular and molecular mechanisms related to ALS pathogenesis (Hirth, 2010; Casci and Pandey, 2015). We utilised the Gal4/UAS system (Brand and Perrimon, 1993) for targeted expression and disease-related accumulation of the *Drosophila* homologue of KPNA4, Importin-α3 (Impα3) (Mason and Goldfarb, 2009). We first investigated its effect on the nuclear-cytoplasmic localisation of the *Drosophila* TDP-43 homolog TBPH (Diaper et al., 2013) and used the *FKH-Gal4* driver to overexpress *UAS-Impα3* in salivary gland cells that are large in diameter with a distinct nuclear to cytoplasm ratio that can be readily assessed (Solomon et al., 2018). To distinguish between the nucleus and cytoplasm, we co-immunolabelled with DAPI (nuclear stain) and anti-MAB414 which recognises nuclear pore complex proteins and immunolabels the nuclear rim (Davis and Blobel, 1987). Immunolabelling of anti-Importin-α3 in *FKH-Gal4* flies crossed with UAS-Impα3 expressing flies (*FKH>Impα3*) revealed an increase in nuclear expression of *Impα3* (Figure 4A) and an increased nuclear-cytoplasmic ratio of Importin-α3 (Figure 4B) when compared to control background (*FKH/+* heterozygous control vs. *FKH>Impα3*; Tukey *t*-test: *p* = 0.0068).

Next, we expressed TBPH with a mutated nuclear localisation signal (TBPH-ΔNLS) under control of the endogenous *TBPH* promotor in an otherwise wildtype background expressing two copies of endogenous *TBPH* (Solomon et al., 2018). When compared to *FKH/+* heterozygous and *w^1118^* control flies, anti-Importin-α3 immunolabelling revealed the cytosolic mislocalisation and near-complete nuclear depletion of Impα3 (Figure 4A; asterisk). Notably, confocal microscopy analysis also revealed that Importin-α3 accumulated around the nuclear rim (Figure 4A; arrowheads). The nuclear loss of Importin-α3 could be partially reverted in *ΔNLS; FKH>Impα3* flies by overexpression of Importin-α3 (Figure 4A). Quantitative assessment revealed Impα3 overexpression increased the nuclear ratio of Importin-α3 in *ΔNLS; FKH>Impα3* flies, when compared to *TBPH-ΔNLS* flies (Figures 4A,B; Tukey *t*-test: *p* = 0.0012) which, however, was markedly lower than in both *FKH/+* and *w^1118^* controls (Figures 4A,B; Tukey *t*-test: *p* < 0.0001). Together these data demonstrate that cytoplasmic accumulation of TBPH causes Importin-α3 pathology in *Drosophila*.

### Accumulating *Impα3* does not mislocalise but partially restores nuclear TBPH

To determine whether accumulating Importin-α3 can cause TBPH pathology, we carried out anti-TBPH immunolabeling in *FKH>Impα3* flies compared to *w^1118^* and *FKH/+* controls. Overexpression of Importin-α3 did not cause any obvious cytoplasmic mislocalisation of TBPH (Figure 4C). However, quantitative assessment of the nucleocytoplasmic ratio of TBPH revealed a statistically significant increase of nuclear TBPH compared to *w^1118^* and *FKH/+* controls (Figure 4D; Tukey *t*-test: *p* = 0.001 and *p* = 0.0001, respectively). Interestingly, a comparison of MAB414 immunolabelling revealed a largely unaltered pattern between control and experimental genotypes (Figures 4A,C). Together these data demonstrate that cytoplasmic accumulation of TBPH is sufficient to induce Importin-α3 pathology, and not vice versa, and suggest that gain of Importin-α3 can increase TBPH cargo transport into the nucleus.

To test whether gain of Importin-α3 could be used to target TDP pathology, we examined *ΔNLS; FKH>Impα3* flies immunolabelled with anti-TBPH (Figure 4C). The distribution of TBPH was compared against *w^1118^* and *FKH/+* controls, as well as against *FKH>Impα3* and *TBPH-ΔNLS* to investigate their impact on the nuclear-cytoplasmic ratio of TBPH (Figure 4D). *TBPH-ΔNLS* flies exhibited a pronounced cytoplasmic mislocalisation and nuclear depletion of TBPH that was more than twofold lower when compared to *w^1118^* and *FKH/+* controls (Tukey *t*-test: p < 0.0001). Importantly, *ΔNLS; FKH>Impα3* flies immunolabelled with anti-TBPH revealed a nuclear-cytoplasmic ratio significantly greater than in *TBPH-ΔNLS* flies (Figure 4D; Tukey *t*-test: *p* = 0.0398); but noticeably lower than both *FKH/+* and *w^1118^* controls (Figure 4D; Tukey *t*-test: *p* < 0.01). These data suggest that targeted overexpression of Importin-α3 can increase TBPH cargo transport into the nucleus thereby counteracting its cytosolic accumulation and nuclear depletion typical for TDP-43 proteinopathies.

## Discussion

Our findings identify KPNA4 pathology in ALS spinal cord and establish that cytoplasmic accumulation of TDP-43 causes KPNA4 abnormalities in *Drosophila* and human cells. Together with previous observations limited to FTD (Solomon et al., 2018; Chou et al., 2018), our findings thus identify KPNA4 abnormalities as a common denominator and molecular signature of ALS and FTD with TDP-43 pathology.

While the origin and initiating cause of cytosolic TDP-43 accumulation remains enigmatic, recent studies have identified nucleocytoplasmic transport deficits as a key pathogenic mechanism involved in ALS and FTD, especially in C9ALS/FTD (Boeynaems et al., 2016a,b; Kim and Taylor, 2017; Hutten and Dormann, 2020). We and others have demonstrated a direct link between TDP-43 dysfunction and karyopherin abnormalities (Khosravi et al., 2017; Solomon et al., 2018; Chou et al., 2018; Hutten et al., 2020; Hayes et al., 2020). These studies revealed that karyopherins, in particular importins/karyopherin alphas (Lee et al., 2016; Khosravi et al., 2017; Hutten et al., 2020), are sequestered into cytoplasmic inclusions by aggregating *β*-sheet proteins, including fragments of TDP-43 (Woerner et al., 2016; Khosravi et al., 2017). However, whether these karyopherin-related abnormalities are a cause or consequence of TDP-43 pathology has remained elusive (Hutten and Dormann, 2020).

We now present experimental evidence both *in vitro* and *in vivo* that accumulating cytosolic TDP-43 causes the nuclear decrease and cytoplasmic accumulation of KPNA4, cellular phenotypes that also characterise KPNA4 pathology seen in the brain and spinal cord of FTD (Solomon et al., 2018) and ALS patients (herein). In contrast, targeted misexpression of the *Drosophila* KPNA4 homolog Importin-α3 was insufficient to cause cytoplasmic accumulation and nuclear depletion of the TDP-43 homolog TBPH. These data imply that karyopherin abnormalities are a consequence of, rather than initiator of cytoplasmic TDP-43 accumulation. Moreover, our results suggest that an initially modest mislocalisation of TDP-43 can impair the localisation of KPNA4 which in turn impairs nuclear transport, thereby leading to a vicious feedback cycle that further impacts the localisation of TDP-43 as well as nucleocytoplasmic transport in general (Solomon et al., 2018). According to this feedback model, the resulting nucleocytoplasmic transport deficit further exacerbates cytosolic accumulation of TDP-43 and as a secondary consequence also impairs the NPC (Solomon et al., 2018). These data imply that shuttling across the nuclear pore is not only an essential requirement for the proper localisation and function of TDP-43 but also sufficient to prevent its cytoplasmic accumulation and the concomitant mislocalisation of KPNA4. This interpretation is consistent with recent findings showing that a single acetylation-mimetic mutation (K82Q) near the TDP-43 NLS disrupts binding to importins, impairs nuclear import and prevents importin α1/β disaggregase activity, leading to cytoplasmic mislocalization and irreversible aggregation of TDP-43 (Ko et al., 2024).

The functional significance of these karyopherin abnormalities is highlighted by our experiments using *Drosophila*. These experiments show that Importin-α3 is necessary and sufficient for nuclear import of TBPH and that upregulation of Importin-α3 can partially restore nuclear TBPH despite its concurrent accumulation within the cytosol. This incomplete rescue could be attributed to the fact that shuttling of TDP-43 involves both KPNA and KPNB1 which together form a trimeric complex that mediates nuclear import of TDP-43 (Prpar Mihevc et al., 2017). However, a recent study revealed non-significant differences in the nuclear-cytoplasmic ratio of TBPH in *Drosophila* C4da neurons between Importin-α3 overexpression and concomitant overexpression of Importin-α3 and Importin-β1, the *Drosophila* homolog of KPNB1 (Park et al., 2020). In view of our results, this suggests that the lack of Importin-β1 accounts for only a small part of the incomplete rescue of the detected TBPH pathology.

Instead, the observed incomplete rescue is likely attributable to the continued cytoplasmic accumulation of TBPH-ΔNLS. Our results indicate that gain of Importin-α3 can only target endogenous wildtype TBPH which was sufficient to shift the nuclear-cytoplasmic ratio towards the nucleus even in the continued presence of TBPH-ΔNLS. These data imply that upregulating Importin-α3 can restore TBPH transport into the nucleus and thus is able to counteract its nuclear depletion triggered by accumulating cytoplasmic TBPH. These findings are of likely translational significance since familial and sporadic ALS cases do express functional wildtype copies of TDP-43 with an intact NLS that are available for karyopherin-mediated nuclear import (Arai et al., 2006; Neumann et al., 2006; Sreedharan et al., 2008; Kabashi et al., 2008; Yokoseki et al., 2008). Our findings therefore establish KPNA4 as a molecular signature of ALS and FTD and suggest a gain-of- KPNA4 function as a potential therapeutic target that could sustain nuclear TDP-43 essential for cellular homeostasis and neuronal function affected in TDP-43 proteinopathies.

## Data Availability

The original contributions presented in the study are included in the article/Supplementary material, further inquiries can be directed to the corresponding author.

## Footnotes

^1^ https://www.rcsb.org

^2^ https://pymol.org/2/

^3^ http://biocomp.chem.uw.edu.pl/CABSdock

^4^ http://huanglab.phys.hust.edu.cn/hpepdock/

^5^ https://bianca.science.uu.nl/prodigy/

^6^ http://www.yasara.org

^7^ https://discover.3ds.com/discovery-studio-visualizer-download

^8^ https://app.biorender.com
