# Supplementary material for: Accumulation of TDP-43 causes karyopherin-α4 pathology that characterises amyotrophic lateral sclerosis
Source: Front Neurosci. 2025 Jul 23;19:1558227. doi: 10.3389/fnins.2025.1558227 (PMC12325296; doi:10.3389/fnins.2025.1558227)
Supplement: Supplementary file 1 [file Data_Sheet_1.pdf]

SUPPLEMENTARY MATERIAL

Accumulation of TDP-43 causes karyopherin-α4 pathology that characterises amyotrophic lateral sclerosis

Manpreet Singh Atwal[1,*,9], Jerneja Nimac[2,3,9], Urša Čerček[2,3], Sarah Ricarda Goesch[1], Hannah Rebecca Goesch[1], Paraskevi Tziortzouda[1], Tiziana Ercolani[1], Anna Zatorska[1], Terouz Pasha[1], Ivo Carre[1], Jacqueline Mitchell[1], Claire Troakes[4], Bart Tummers[6], Vera Župunski[5], Boris Rogelj[2,5], Tibor Hortobágyi[7,8,‡], and Frank Hirth[1,#]

[#]Correspondence: Frank Hirth; email: Frank.Hirth@kcl.ac.uk

This PDF file includes:

Figures S1-S4

Tables S1 and S2

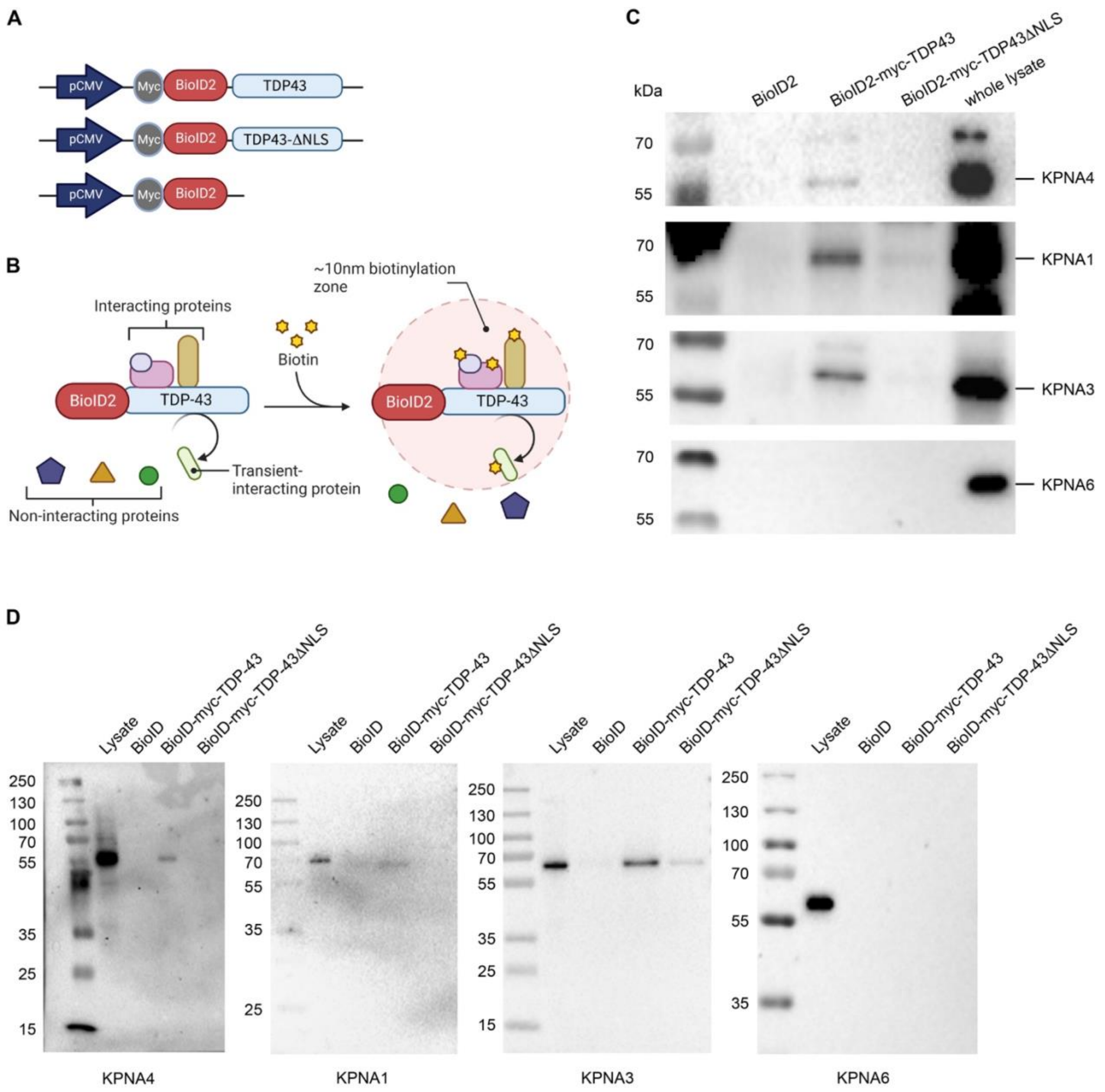

Figure S1. Karyopherin-α interaction with TDP-43 requires nuclear localisation signal. **(A)** Schematic representation of constructs used for stable HEK293 cell lines encoding BioID2 ligase coupled with either wildtype (TDP-43) or nuclear localisation signal (NLS) depleted form of human TDP-43 (TDP-43-ΔNLS). **(B)** Schematic representation of BioID-2 method for detection of proteins in proximity (~10nm) of target protein. **(C)** Western blot of pull-down experiments reveals specific interaction of wildtype TDP-43 with KPNA1, KPNA3 and KPNA4 which is TDP-43-NLS-dependent. BioID2 pulldown did not reveal TDP-43 interaction with KPNA6 or with BioID2-only expressing cells. **(D)** Second independent set of experiments showing entire blots.

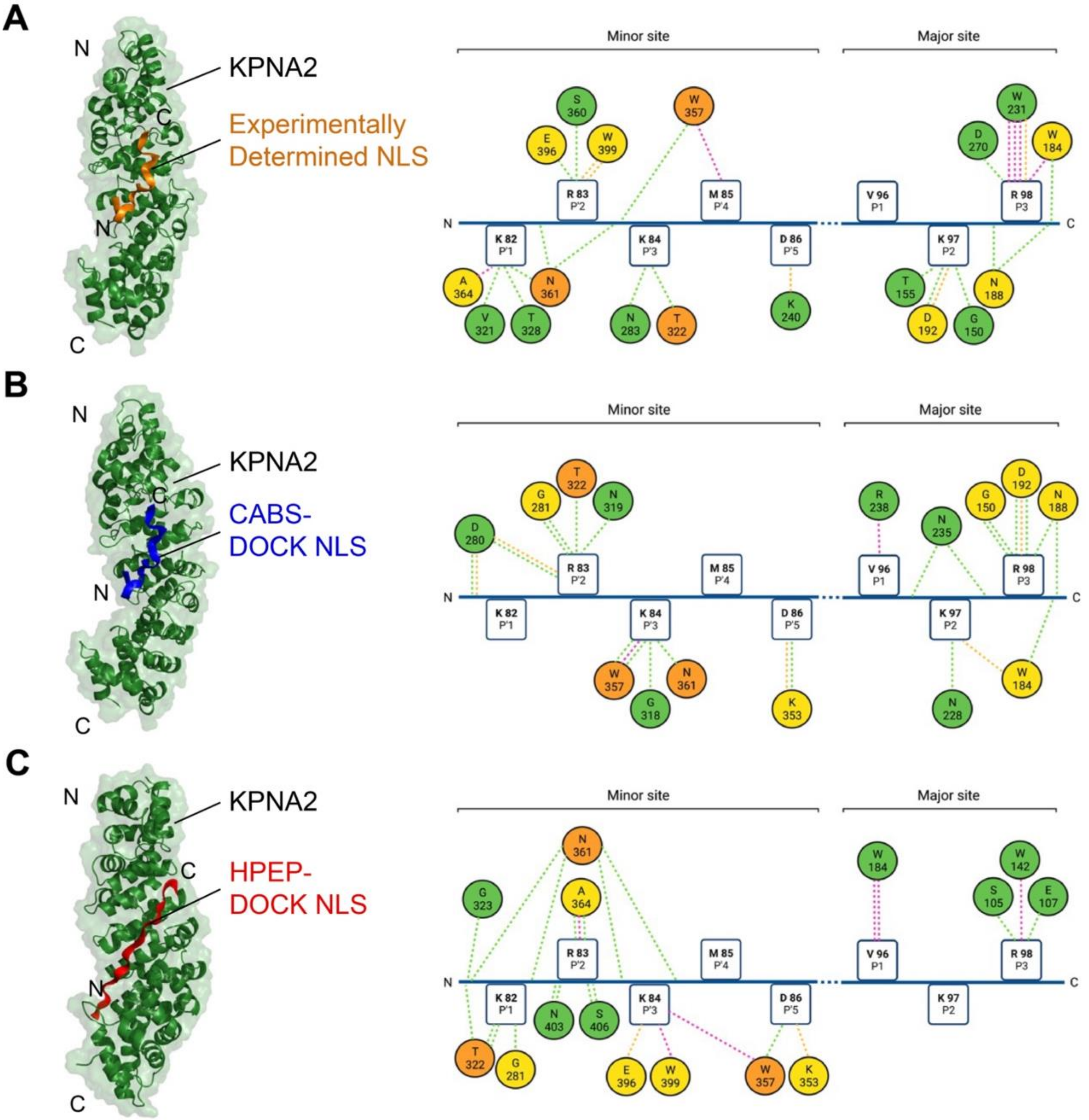

Figure S2. Computational binding prediction of NLS TDP-43 and KPNA2 identifies biophysical interactions corresponding to the experimentally determined model. Structural illustration (left hand side) and interaction contacts of **(A)** experimentally determined **(B)** CABS-DOCK or **(C)** HPEP-DOCK derived binding prediction of NLS TDP-43 and KPNA2 (PDB: 7N9H) with the corresponding molecular contact maps (right hand side). KPNA2 residues engaged with the minor and major site of NLS TDP-43 (squares) are depicted either in orange (present in all three models), yellow (present in two models) or green (present in one model) spheres. Interaction types are colour coded: electrostatic interactions and salt bridges (orange), hydrogen bonds (green) and hydrophobic interactions (pink). Contact maps were created with Biorender.com.

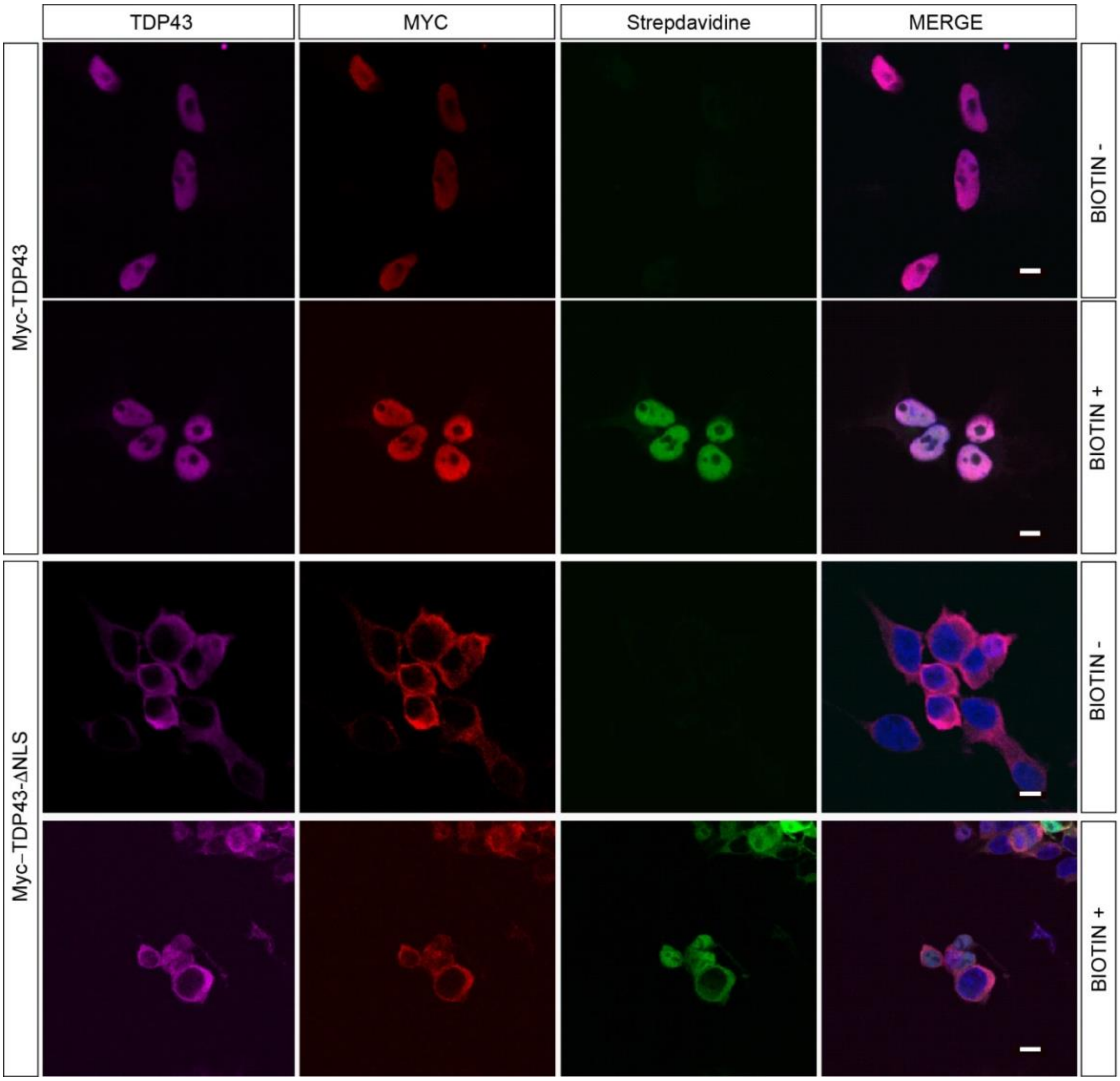

Figure S3. Lack of nuclear localisation signal causes cytoplasmic accumulation and nuclear depletion of TDP-43. Analysis of wildtype TDP-43 or TDP-43-ΔNLS expressing cells immunolabelled with anti-TDP-43 (purple) and anti-Myc (red) reveals cytoplasmic accumulation and nuclear depletion of TDP-43 in the absence of a functional nuclear localisation signal (NLS). Streptavidine detection (green) revealed that BioID2 ligase was only active in the presence of 50 µM biotin. Note, biotinylated proteins locate in the same area as construct expressing TDP-43 or TDP-43-ΔNLS. Scale bars, 10 µm.

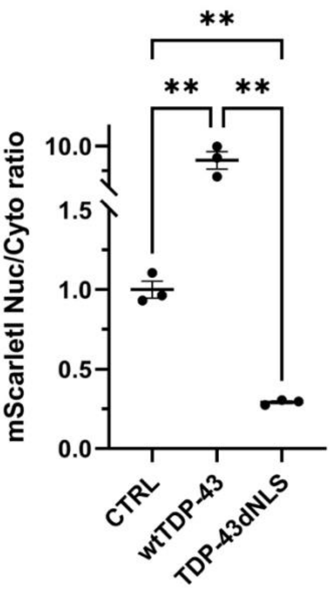

Figure S4. Nucleocytoplasmic ratio of mScarletI-myc in examined conditions. Control experiment quantifying the nuclear-cytoplasmic ratio of mScarletI-myc expressed either on its own (CTRL; n = 831) or fused to full-length human wildtype TDP-43 (mScarletI-myc-wtTDP-43; n = 826) or fused to the NLS-deleted from of human TDP-43 (mScarletI-myc-TDP-43ΔNLS; n = 1081). Expressed on its own, mScarletI-myc is equally distributed in the nucleus and cytoplasm; however, its subcellular distribution was significantly increased in the nuclei when fused to wt-TDP-43 and significantly decreased when fused to TDP-43ΔNLS, consistent with the localisation of the TDP-43 variant. Statistical analysis was performed using one-way ANOVA with Tukey's multiple comparison post-hoc test; ** $p<0.01$; mean ± SEM shown.

COMPUTATION	PARAMETER	KPNA2
**CAPS-DOCK**	Electrostatic interactions	9
	Hydrogen bonds	30
	Hydrophobic interactions	6
**HPEP-DOCK**	Electrostatic interactions	6
	Hydrogen bonds	34
	Hydrophobic interactions	10
**Experimental (7N9H)**	Electrostatic interactions	7
	Hydrogen bonds	20
	Hydrophobic interactions	13

Supplementary Table S1. Binding characteristics of computed and experimental KPNA2-TDP-43-NLS complex. Parameters determined with CAPS-DOCK and HPEP-DOCK computation, compared to experimental structure 7N9H.

COMPUTATION	PARAMETER	KPNA1	KPNA4	KPNA6
CAPS-DOCK	Electrostatic interactions	15	5	10
	Hydrogen bonds	36	20	24
	Hydrophobic interactions	10	14	3
	Molecular contact surface area (Å^2)	1,226.6 Å^2	994.4 Å^2	916.5 Å^2
	Binding energy (kcal/mol)	-11.5	-11.4	-9.6
	Dissociation constant	$3.4E^{-09}$	$4.6E^{-09}$	$8.7E^{-08}$
HPEP-DOCK	HPEP-DOCK docking score	-187.2	-183.2	-181.9
	Binding energy (kcal/mol)	-10.5	-10.1	-10.0
	Dissociation constant (M)	$1.9E^{-08}$	$3.8E^{-08}$	$4.9E^{-08}$

Supplementary Table S2. Binding characteristics of computed KPNA-TDP-43-NLS complex. Parameters determined with CAPS-DOCK and HPEP-DOCK computation for KPNA1, KPNA4 and KPNA6.
